# AML/Normal Progenitor Balance Instead of Total Tumor Load (MRD) Accounts for Prognostic Impact of Flowcytometric Residual Disease in AML

**DOI:** 10.3390/cancers13112597

**Published:** 2021-05-26

**Authors:** Diana Hanekamp, Jesse M. Tettero, Gert J. Ossenkoppele, Angèle Kelder, Jacqueline Cloos, Gerrit Jan Schuurhuis

**Affiliations:** 1 Department of Hematology, Amsterdam University Medical Centers, Cancer Center VU University Medical Center, 1081 HV Amsterdam, The Netherlands; d.hanekamp@erasmusmc.nl (D.H.); j.tettero@amsterdamumc.nl (J.M.T.); g.ossenkoppele@amsterdamumc.nl (G.J.O.); a.kelder@amsterdamumc.nl (A.K.); j.cloos@vumc.nl (J.C.); 2 Department of Hematology, Erasmus MC, NL-3000 CA Rotterdam, The Netherlands

**Keywords:** acute myeloid leukemia, minimal/measurable residual disease, multiparameter flow cytometry, primitive compartment, prognostic value, MRD false negativity, MRD false positivity

## Abstract

**Simple Summary:**

Measurable residual disease (MRD), taken as the percentage of white blood cells in acute myeloid leukemia, has important prognostic value, but false negatives and false positives can occur. Immature populations make up the most important part of MRD (now referred to as WBC-MRD). We explored the influence on prognostic impact of the two compartments of WBC-MRD: (1) the AML part of the total primitive/progenitor (CD34+, CD117+, CD133+) compartment (primitive marker MRD; PM-MRD) and (2) the total progenitor compartment (as % of WBC, PM%). Both are related as follows: WBC-MRD = PM-MRD × PM%. In the HOVON/SAKK study (H102; *n* = 300), using two objectively assessed cut-off points (2.34% and 10%), PM-MRD was found to be prognostically more discriminative than WBC-MRD. The PM% parameter had no prognostic impact and, moreover, resulted in WBC-MRD false positives/false negatives. Highly important for present clinical practice is the identification of a PM-MRD ≥ 10% but MRD^negative^ (MRD < 0.1, ELN consensus) poor prognosis subgroup. This suggests that a residual disease analysis using PM-MRD should be conducted.

**Abstract:**

Measurable residual disease (MRD) in AML, assessed by multicolor flow cytometry, is an important prognostic factor. Progenitors are key populations in defining MRD, and cases of MRD involving these progenitors are calculated as percentage of WBC and referred to as white blood cell MRD (WBC-MRD). Two main compartments of WBC-MRD can be defined: (1) the AML part of the total primitive/progenitor (CD34+, CD117+, CD133+) compartment (referred to as primitive marker MRD; PM-MRD) and (2) the total progenitor compartment (% of WBC, referred to as PM%), which is the main quantitative determinant of WBC-MRD. Both are related as follows: WBC-MRD = PM-MRD × PM%. We explored the relative contribution of each parameter to the prognostic impact. In the HOVON/SAKK study H102 (300 patients), based on two objectively assessed cut-off points (2.34% and 10%), PM-MRD was found to offer an independent prognostic parameter that was able to identify three patient groups with different prognoses with larger discriminative power than WBC-MRD. In line with this, the PM% parameter itself showed no prognostic impact, implying that the prognostic impact of WBC-MRD results from the PM-MRD parameter it contains. Moreover, the presence of the PM% parameter in WBC-MRD may cause WBC-MRD false positivity and WBC-MRD false negativity. For the latter, at present, it is clinically relevant that PM-MRD ≥ 10% identifies a patient sub-group with a poor prognosis that is currently classified as good prognosis MRD^negative^ using the European LeukemiaNet 0.1% consensus MRD cut-off value. These observations suggest that residual disease analysis using PM-MRD should be conducted.

## 1. Introduction

In the treatment of acute myeloid leukemia (AML), the assessment of measurable residual disease (MRD) is of high importance. Nowadays, MRD is assessed with molecular methods (PCR and next-generation sequencing [1,2,3,4]), and for the majority of cases, multicolor flow cytometry (MFC), which has been found to have high prognostic value for disease outcomes in many studies [5,6,7,8,9,10,11,12,13,14,15]. MFC-MRD is classically defined as the number of cells with a leukemic associated immunophenotype (LAIP), usually expressed as the percentage of the complete white blood cell (WBC) population. European LeukemiaNet (ELN) recommendations define MRD positivity as ≥0.1% LAIP^+^ cells/WBC [12]. Apart from the approach that uses LAIPs established at the time of diagnosis, ELN promotes the so-called difference from normal approach, which assesses aberrant differentiation bone marrow patterns, with subsequent quantification of aberrancies or no quantification (“any MRD is MRD positive”) [16,17].

While MFC is the major method used to assess MRD, variation among laboratories is high [18], and several attempts have been made to improve harmonization [19,20,21]. Therefore, there is a need for harmonization and, wherever possible, standardization of assays. Whereas standardization for sample acquisition, shipment, and storage can be achieved by strict rules [22,23,24,25], standardization in the analysis and reporting of MRD remains a challenge [12].

A major criticism of MFC-MRD is that it is relatively subjective, since it requires extensive knowledge of normal bone marrow (NBM) differentiation patterns [12]. In addition, the background of LAIP antigens on normal cells may lead to low LAIP specificity and therefore low sensitivity in the detection of specific, i.e., AML defining, LAIPs [26,27,28]. This is especially important at lower MRD levels, where background levels may approach patient MRD levels. It also has to be emphasized that LAIPs may change during/after therapy, resulting in the disappearance of populations or emergence of new populations [29,30,31].

Recently, we postulated an alternative MRD approach that has the potential to simplify or better standardize MFC-MRD [26], because it allows one to quantitatively and more objectively assess MRD. It is known that CD34-positive, CD117-positive, and CD133-positive leukemia compartments contain normal progenitors and leukemia initiating and propagating cells [32,33,34]. We hypothesized that the relative contribution of AML progenitors within the total progenitor compartment (AML + normal) may be equally informative for relapse initiation as the total leukemic load, which, in turn, represents all aberrant cells and is usually expressed as percentage of WBC. If non-dividing more mature cells are included, this is referred to as MFC-MRD, and when only progenitors are included, it is referred to as WBC-MRD. Indications for a prognostic impact of AML progenitors among the total progenitor compartment were previously reported in a small study [35].

In this paper we show that, for WBC-MRD, it may not be the total tumor load (WBC-MRD%) that has the main prognostic impact on AML, but rather, the contribution of the AML progenitor part of the total progenitor population (referred to in this paper as the primitive marker MRD; PM-MRD). Moreover, we identify a factor that is not only superfluous but may even abrogate the prognostic impact of WBC-MRD in some patients. 

## 2. Materials and Methods

The present study was designed to test a new MRD-related hypothesis based on data obtained during a previous published trial [36,37]. This MRD hypothesis was compared to an MRD assessment reported in a relevant publication [37]. An additional five patients were excluded from the original analysis because PM-MRD data were missing.

Immunophenotyping was performed as previously described [38]. Flow cytometry was performed on FACS CANTO (BD Biosciences, San Jose, CA, USA) with either 6- or 8-color antibody panels (see Table S2 in Zeijlemaker, et al. [37]). To be able to fully compare the contributions of the two parameters (PM-MRD and PM%) with the prognostic impact of MRD, we used the data assessed in the Zeijlemaker study. All other relevant data used to calculate MRD (WBC, CD34, or CD117 or the CD133 percentage) were assessed from the tube used for the MRD calculation in that study.

Analyses of flow cytometric measurements were performed with Infinicyt^™^ software (Cytognos, Salamanca, Spain). The LAIPs used in this study and the frequencies of use are shown in Table S1. Sampling for clinical MRD assessment was done after the second induction therapy and after hematologic recovery. The percentage of the population with the primitive marker (necessary for WBC-MRD) therefore approximately reflects bone marrow (BM) steady state. A retrospective analysis revealed nine patients who had relapsed at the time of sampling after the second induction. Since these patients were included in all previously published papers on this dataset, they were not excluded. Furthermore, our analyses showed that the important PM-MRD and WBC-MRD cut-off values used in the present study did not change upon exclusion of the nine relapsed samples, i.e., the later defined cut-off values of 2.34% and 10% for PM-MRD remained exactly the same, as did the MRD cut-off of 0.1%, while the MRD cut-off of 0.03% changed only slightly (down to between 0.02% and 0.03%).

The WBC-MRD assessment in the present study was based on the LAIP approach [37]. Gating was performed following strict criteria concerning the forward/side scatter properties of the LAIP, the expression pattern of CD45, primitive marker (PM) expression (i.e., CD34, CD117, and/or CD133), and the expression of a myeloid marker (i.e., CD13, CD33, and/or HLADR) to exclude aspecificity and non-relevant cell types (extensively described in Cloos, et al. [23] and Zeijlemaker, et al. [39]). To discriminate between LAIP+ and LAIP- on cells, their expression levels on lymphocytes were used together with the knowledge that blasts usually have higher autofluorescence.

**Figure 1:**
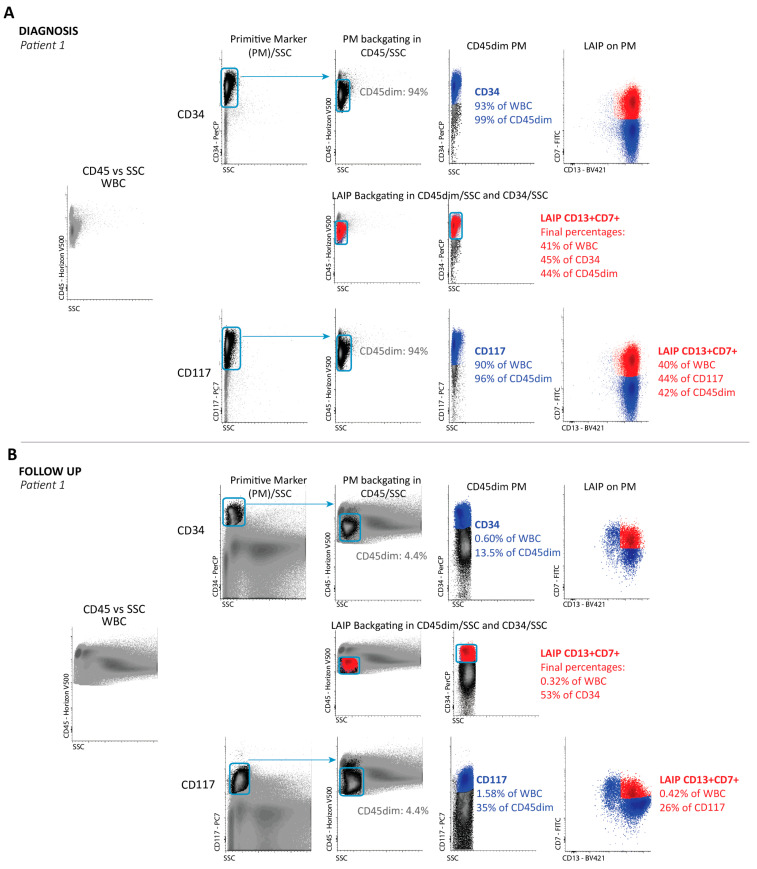

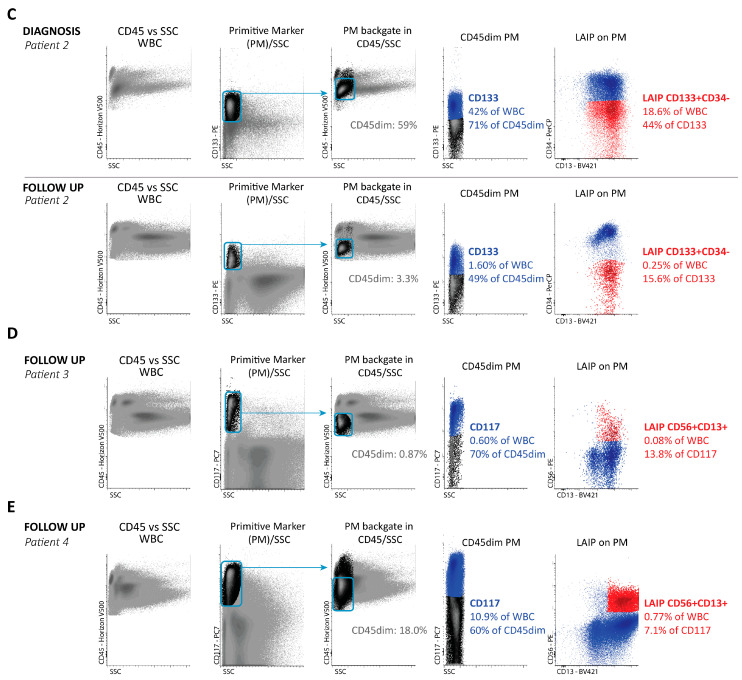
Flow cytometric examples of WBC-MRD/PM-MRD. Gating is described in more detail by Cloos, et al. [23] and Zeijlemaker, et al. [39]. (**A**) Gating of progenitor populations and LAIPs at the time of diagnosis (diag) in one patient (patient 1). First panel: WBC in CD45/SSC; first row: gating for CD34 and CD34+ LAIPs; second row: sequential backgating in CD45dim/SSC and CD34/SSC of the LAIP+ population—this is only shown for this CD34 LAIP, but this sequential backgating of LAIP+ populations was performed in all cases (so also below for CD117 LAIPs and CD133 LAIPs) in CD45dim/SSC and PM/SSC and, where necessary, also in FSC/SSC; third row: gating for CD117. (**B**) Gating on patient 1 at follow-up (after second induction course). First panel: WBC in CD45/SSC; first row: gating for CD34 and CD34+LAIPs; second row: sequential backgating in CD45dim/SSC and CD34/SSC of the LAIP+ population—again, this is only shown for CD34, but was applied for all other follow up samples in (**B**–**E**); third row: gating for CD117 and CD117+ LAIPs. (**C**) The same data (without backgating) as shown in Figure 1A,B are provided but for another patient with the CD133+/CD34- LAIP at diagnosis (first row) and at follow-up (second row). LAIP+ percentages in all cases (**A**–**C**) reflect the situation after the extra backgating steps were conducted. (**D**) Identification of a CD56+ LAIP at follow-up in a patient (patient 3) with no diagnosed LAIPs. MRD was relatively low (0.08%), but PM-MRD was relatively high (13.8%); these are characteristics of group IV in Figure 4D. (**E**) Example of a patient (patient 4) at follow-up with a high PM% (CD117%: 10.9%), and high MRD (0.77%) but with an intermediate PM-MRD (7.1%); these are characteristics of group V in Figure 4D.

Examples of gating of CD34+, CD117+, and CD133+ PM cells, both at diagnosis and at follow-up, are shown in Figure 1. In a separate analysis, we found that, with a few exceptions, the maximal background of LAIP expression on progenitors in NBM was <1%, and for CD133^+^CD34^−^ LAIPs, it was <0.2% [40]. For WBC-MRD, the value was <0.02%. We were thus able to safely use uncorrected LAIP+ values for PM-MRD and WBC-MRD. CD34 was used in most LAIPs in CD34 positive AML. CD133 was particularly used in LAIPs in CD34low/neg AML cases. CD133 is a marker of more immature CD34+ cells and covers about 50% of BM CD34+ cells [41]. We used CD133 because of its very low background. CD117 may well be able to replace CD133 in most cases. In a larger survey of 858 patients in several HOVON studies, the CD133+CD34^−^ LAIP was used 45 times (5%). In 34 of these cases, a CD117 LAIP could have been used instead, leaving only 12 patients (1.4% of the total patient population) in which the CD133+CD34^−^ LAIP was the only option. This shows that in over 98% of cases, CD34 and/or CD117 LAIPs enable the analysis of WBC-MRD and PM-MRD. We further made an inventory of possible triple negative (CD34neg/CD133neg/CD117neg) cases in which the definition of progenitor aberrancies would be impossible. We studied all 858 patients from the whole HOVON study. The expression cut-off of 1% positivity for CD34 and CD133 defines patients for whom defining AML-related aberrancies below this cut-off is impossible [42,43,44]. CD34 negativity was found in 73/858 cases; CD34/CD133 double negativity in 15 out of these 73 CD34 negative cases. In all of these 15 cases, CD117 expression was high (mostly >10%; 3.5% at least). So, in none of the 858 cases did triple negativity occur.

MFC-MRD is used to define residual disease in the classical way (golden standard), which includes aberrant populations (progenitors + non-progenitors) as a percentage of all WBC (see Supplementary Text S1 for precise definition). 

In our institute, under MRD conditions, the incorporation of more mature populations was not found to have major effects on MRD. Only a few patients were differently characterized as being MRD positive when more mature cells were included, and therefore WBC-MRD (AML progenitors/WBC) forms the basis of the current paper. We dissected WBC-MRD into two components as follows:

1. The “total PM population” (which is the sum of LAIP^+^ AML progenitors and LAIP^-^ normal progenitors) and is expressed as the percentage of WBC (now referred to as **PM%**),

2. The contribution of LAIP^+^ AML progenitors to the total (LAIP^+^ + LAIP^-^) progenitor compartment. In this paper, this LAIP^+^ compartment is referred to as **PM-MRD** (which ranges from a ratio of 0 (0% of cells are LAIP^+^) to 1.0 (100% of cells are LAIP^+^). Thus, 

**PM-MRD = LAIP^+^-progenitors/ (LAIP^+^ + LAIP^−^)-progenitors.**

The relationship between PM% and PM-MRD is: 

**WBC-MRD = PM% × PM-MRD.**

An example of a calculation is shown in Supplementary Text S1, and examples of flow cytometry are shown in Figure 1. It should be mentioned that, in this paper, the prognostic impact of PM-MRD is primarily compared with the optimal prognostic impact of WBC-MRD using the progenitors used in a previous manuscript detailing the HOVON/SAKK study H102 data [37]. In practice, this means that, in some cases, WBC-MRD is corrected for the partial coverage of diagnosis progenitors with LAIP [26]. In this paper, we analyze the contributions of both components (PM-MRD and PM%) to the prognostic impact of WBC-MRD. 

In survival Kaplan–Meier analyses, we used event free survival (EFS), which is defined as the time between sampling after complete remission (CR) and the date of relapse/progressive disease or death. Patients with no recorded event were censored at the date of last follow-up. Kaplan–Meier analyses were performed using the survival R package. Statistical analyses were performed using SPSS version 22.0 software. Outcomes between groups were compared using the log-rank test. The MaxStat package of R identified optimal cut-off point(s) for PM-MRD by calculating the LogRank for every possible PM-MRD value.

The prognostic values of WBC-MRD (cut-off 0.03% and 0.1%) and PM-MRD (cut-off 2.34% and 10.0%) for EFS were investigated in multivariable models including variables that were significant in univariate analyses.

## 3. Results

### 3.1. Calculation of the PM-MRD Cut-Off Value Compared to WBC-MRD

**Figure 2:**
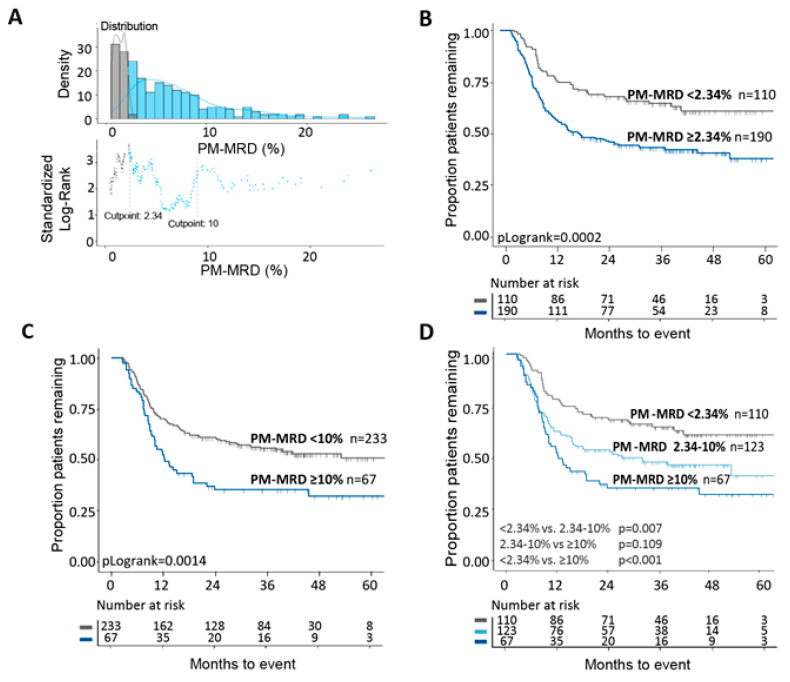
Prognostic impacts of different PM-MRD cut-off values. (**A**) Using the standard Log-Rank analysis, the optimal cut-off values of 2.34% and 10.0% were determined (X-axis denotes % PM-MRD). (**B**) Using the cut-off value of 2.34% allowed the discrimination of two patients groups with different EFS: 42.6% for PM-MRD^positive^ and 63.6% for PM-MRD^negative^. (**C**) For the 10.0% cut-off value, EFS was 34.3% for PM-MRD^positive^ and 54.9% for PM-MRD^negative^. (**D**) When the two were combined, three patient groups with different EFS rates were identified: 63.6%, 47.2%, and 34.3%.

**Figure 3:**
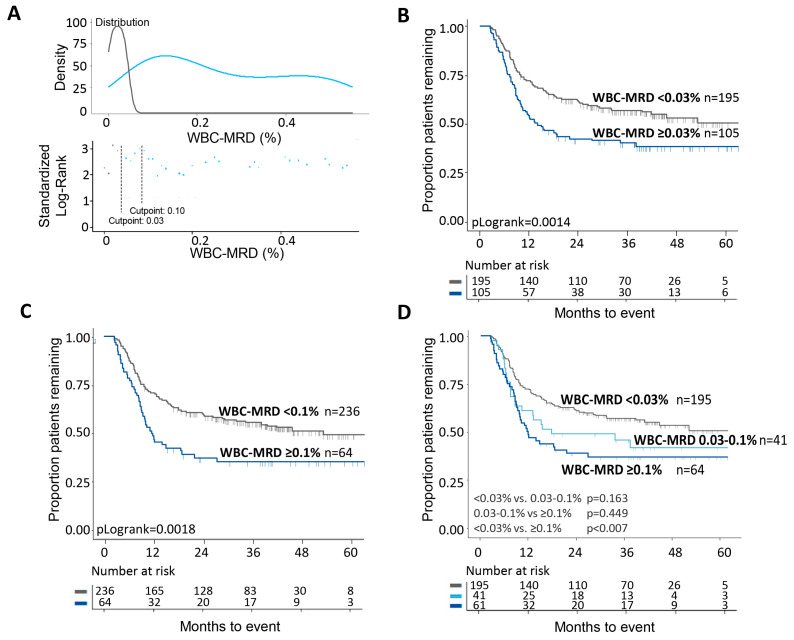
Prognostic impact of different WBC-MRD cut-off values. (**A**) Using MaxStat analysis, optimal cut-off values of 0.03% and 0.1% were identified (X-axis denotes % WBC-MRD). In contrast to PM-MRD, the cut-off points are not that clear, especially for a cut-off value of 0.1%. (**B**) Using the cut-off of 0.03%, two patient groups showed an EFS of 40.0% for WBC-MRD^positive^ patients and an EFS of 55.9% for WBC-MRD^negative^ patients. (**C**) For the 0.1% cut-off values, these groups showed an EFS of 37.5% for MRD^positive^ patients and an EFS of 53.8% for MRD^negative^ patients. (**D**) When both were combined, three patient groups with different EFS were shown: 55.9%, 43.9%, and 37.5%.

To determine the prognostic value of PM-MRD, MaxStat statistics was used to define cut-off levels that can distinguish patients with different EFS. There were two optimal cut-off values (2.34% and 10%; Figure 2A) that each allowed the identification of two distinct patient populations with different 5-year EFS (Figure 2B,C). Together, the two cut-off values thus allowed discrimination among three patient groups with different EFS: 63.6%, 47.2%, 34.3% (Figure 2D). For WBC-MRD, as used in our previous study [37], MaxStat cut-off values were less pronounced, but a cut-off level of 0.03% was identified. We further analyzed the ELN consensus cut-off value of 0.1% (Figure 3A). Again, these two WBC-MRD cut-off values each identified two distinct patient groups (Figure 3B,C). The EFS differences between the groups were less pronounced compared with PM-MRD: there were three patient groups with 5-year EFS values of 55.9%, 43.9%, and 37.5% (Figure 3D). Additional cut-off values, for both PM-MRD and WBC-MRD, did not identify patient groups with better or worse survival rates that reached significance (groups were too small). Univariate and multivariate cox regression analyses (Tables S2 and S3) showed that PM-MRD and WBC-MRD are both independent prognostic factors. Together with Figure 2 and Figure 3, the data suggest that there is better discrimination between patient sub-groups for PM-MRD. 

Previously we showed that the 0.1% WBC-MRD cut-off value defines patient groups with different outcomes in different cytogenetic risk groups [38]. Similarly, the use of both PM-MRD cut-off values allows three patient groups with different survival rates to be defined, especially within the intermediate HOVON risk group (where MRD may be used to guide the use of consolidation treatment [15,45]) and in the poor risk group. (Figure S1A–C). For comparison, WBC-MRD (0.03% and 0.1% cut-off values) is shown in Figure S1D–F. 

It may be argued that cases of more mature AML may not be suitable for analysis via the progenitor approach. However, our cohort included 46 mature AML cell patients (15.3%), classified as French–American–British (FAB) classifications M5 (*n* = 38), M6 (*n* = 6), or M7 (*n* = 2), which all had primitive compartments that could be evaluated for LAIP expression. PM-MRD and WBC-MRD (cut-off values at 10% and 0.1%, respectively) allowed us to identify patient groups with distinct different EFS (pLogrank = 0.001 and 0.009, respectively) (Figure S2B,D).

### 3.2. Role of AML/Normal Progenitor Load versus Total Tumor Load

**Figure 4:**
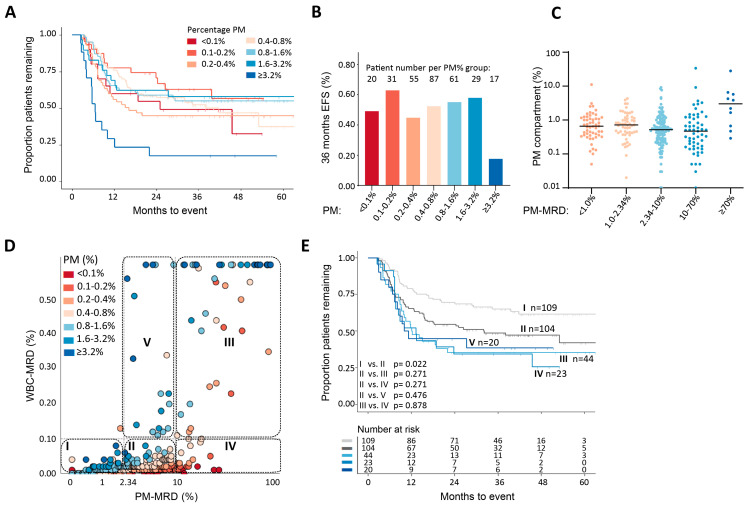
Role of the percentage of the whole (AML + normal) PM compartment (“PM%”) in the combined prognostic value of PM-MRD and WBC-MRD. The PM% in the total patient group was subdivided as shown in A (inset). (**A**) The Kaplan–Meier analysis showed that there was no consistent difference in EFS between patients in different PM% groups, except for the group with PM% ≥ 3.2% (lower EFS), but in this group, 4/17 samples were from patients who had already officially relapsed at the time of sampling. (**B**) EFS at 36 months in the different PM% groups. No consistent increase or decrease in EFS was seen with an increasing PM%, except in the PM ≥ 3.2% sub-group. (**C**) Distribution of PM% within different PM-MRD regions. Large heterogeneity of PM% was found across the whole range of PM-MRD in the group of patients as a whole. (**D**) Relationship between WBC-MRD and PM-MRD. The correlation between PM-MRD and WBC-MRD was moderate (r = 0.737, *p* < 0.001). In sub-group IV, (very) low PM percentages (identified by the different reddish/orange colors) result in (very) low MRD values (because PM-MRD × PM% will be <0.1), despite the high, prognostically unfavorable PM-MRD values (≥10). When no correction of WBC-MRD for LAIP coverage at diagnosis was applied (so WBC-MRD^not corrected^), the results were only altered slightly. This is further explained in Supplementary Text S1. These results confirm the strength of the prognostic impact of the PM-MRD ≥ 10% group. (**E**) Analysis of EFS in sub-groups defined by PM-MRD cut-off values of 2.43% and 10% and by a WBC-MRD cut-off value of 0.1%. Sub-group V is considered in Texts S1 and S2. The cytogenetic and molecular characteristics of the five subgroups can be found in Table S4.

PM-MRD and WBC-MRD both show prognostic value; however, the survival data only show partial overlap with PM-MRD exhibiting more a favorable prognostic impact (Figure 2 and Figure 3, Table S3). The only difference between them is the PM% (see Materials and Methods). Although PM% is a major determinant of the total tumor load (the difference between the patient with the lowest PM%, <0.01%, and highest PM%, >10%, is a factor >1000), except for the group with the highest values, i.e., PM% ≥ 3.2%, no correlation with EFS was found in the univariate analysis (Table S2). In the multivariate analysis (Table S3), the prognostic impact of WBC-MRD was therefore found to be independent of PM%. To illustrate this in a more practical way, the whole patient group was divided into sub-groups with ranges of PM% differing by a factor of 2 (Figure 4A, inset and Figure S3). In line with the univariate and multivariate analyses, this separation did not result in consistent differences in EFS between the groups in the Kaplan–Meier analysis (Figure 4A) or the EFS at 36 months (Figure 4B), except for in the ≥3.2% PM% group. These results suggest that, in the equation WBC-MRD = PM% × PM-MRD, the prognostic impact of WBC-MRD likely originates mainly from the PM-MRD component.

### 3.3. Differences in Prognostic Impact between PM-MRD and WBC-MRD

**Figure 5:**
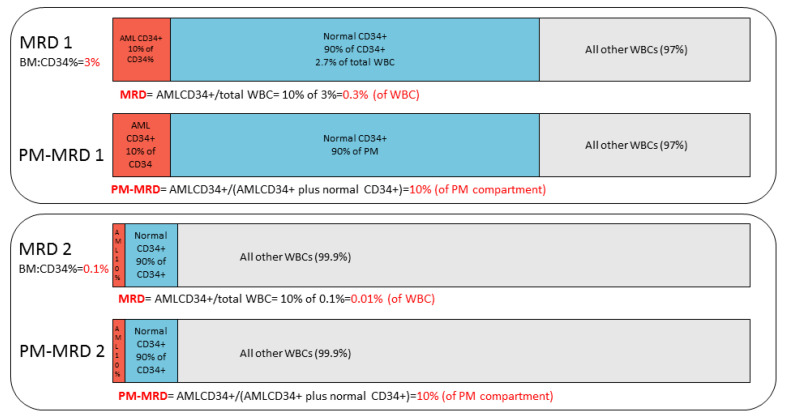
Disturbed relationship between WBC-MRD and PM-MRD. PM-MRD and WBC-MRD positivity in a schematic example of 2 clinical samples differing in terms of CD34 percentage. In example 1, the AML CD34+ cells (in brown) make up 10% of the total CD34+ compartment (brown + blue). Here, PM-MRD is thus 10%. Since the BM CD34+ percentage in this example was chosen to be 3% (of WBC), the AML part of the total WBC population, representing WBC-MRD, was 10% of 3% = 0.3%. In example 2, the PM-MRD was again 10% (in brown), but since the BM CD34+ percentage (brown + blue) in this example was 0.1% (of WBC), the AML part of the total WBC population (WBC-MRD) was 10% of 0.1% = 0.01%. These examples of the most extreme conditions of BM CD34% show that samples 1 and 2 have similar PM-MRD (10% of CD34+ compartment), suggesting that they have similarly poor prognoses (see Figure 2D). On the contrary, sample 1 has a high WBC-MRD (0.3%), suggesting a poor prognosis, but sample 2 has a low WBC-MRD (0.01%), suggesting a good prognosis.

To better understand the prognostic differences between PM-MRD and conventional WBC-MRD, we plotted their values for all individual patients (Figure 4D). Although, overall, a correlation (r = 0.737, *p* < 0.001) was found, there were substantial differences. The clinically most important consequences for the present application of MRD concern the prognostic meaning of an (ELN consensus) MRD cut-off value of 0.1%. As shown in Figure 4D,E, sub-group IV (PM-MRD ≥ 10% (PM-MRD^positive^)/MRD < 0.1% (MRD^negative^)) and sub-group III (PM-MRD ≥ 10% (PM-MRD^positive^)/MRD ≥ 0.1% (MRD^positive^) were shown to have similarly poor prognoses. As a consequence, the present ELN consensus MRD cut-off value of 0.1% [12] cannot identify patients (*n* = 23) within compartment IV as being of poor risk. This shows that the inclusion of the PM% in patients with high PM-MRD results in MRD false negatives. This concept of the PM% interfering with the prognostic impact of the PM-MRD compartment is illustrated schematically in Figure 5 for two patients with the same high PM-MRD but with different (a low and a high) PM% values. The relationship between PM-MRD and MRD is shown and further analyzed in more detail in Supplementary Text S2 and Figure S4.

Lastly, we studied whether it is possible to carry out the PM-MRD approach in a different-from-normal setting. Figure 1C shows an example of PM-MRD (and WBC-MRD) in the absence of diagnosed LAIP. In this case, the sample has the characteristics of a group IV patient, i.e., with high PM-MRD (>10%) but low MRD (<0.1). In a separate data set, we picked patient data from an arbitrary time period in the HOVON102 study. The files for these patients were studied by flow cytometrists who blindly (i.e., without knowledge of diagnosis information that was, however, available for later comparison) evaluated follow-up samples to identify possible WBC-MRD cases and their corresponding PM-MRD values (Table S5A). This approach allowed a direct comparison with the “Diagnosis LAIP approach”, as applied in the clinical study reported in the present paper. We found that all LAIPs (both as WBC-MRD and as PM-MRD), as “blindly” identified in follow-up testing, matched the LAIPs found earlier at diagnosis and seen in the follow-up testing in the clinical study. Moreover, in some cases, extra LAIPs were found (both defining WBC-MRD and PM-MRD), suggesting that other populations emerged in remission (perhaps reflecting different “clones”). In another small dataset, examples are shown of follow-up LAIPs (both WBC-MRD and PM-MRD) identified in the complete absence of diagnosis information (Table S5B).

## 4. Discussion

In almost all studies on MRD in AML, MRD is defined as the number of aberrant cells as the percentage of WBC, thereby providing a measure of the total tumor load for a particular patient [5,6,7,8,9,10,11,12]. In this study, we focused on MRD identified using progenitors as done in the clinical HOVON/SAKK 102 study (*n* = 300) [36]. We showed that it is not the total tumor load (WBC-MRD) that accounts for the prognostic impact of MRD, but rather, the AML part of the total progenitor compartment (PM-MRD). PM-MRD was found to be a strong independent prognostic factor, capable of defining three patient groups with different survival rates. Hereby, WBC-MRD and PM-MRD are, by definition, interrelated through the simple formula WBC-MRD = PM-MRD × PM%, in which PM% is the total progenitor compartment as a percentage of WBC.

Furthermore, we showed that PM% is a major factor in the (current) WBC-MRD, but it abrogates the prognostic impact of PM-MRD in some patients by causing an artificially high MRD (due to high PM%) or an artificially low MRD (due to low PM%), which does not reflect the true prognostic impact for the patients involved. For the latter group, this can be most accurately demonstrated for WBC-MRD using the MRD ELN consensus cut-off value of 0.1%. The PM-MRD group (≥10%) with the poorest prognosis not only harbors high WBC-MRD cases with poor prognosis (≥0.1%), but in addition, contains patients with (very) low WBC-MRD values who also have a poor prognosis. In the clinical setting these patients represent WBC-MRD false negative patients. The definition of any MRD cut-off value used to define MRD positivity and MRD negativity for clinical purposes will comprise such false negativity and false positivity issues and will therefore affect clinical decisions.

It needs to be emphasized that WBC-MRD, of course, has prognostic value when comparing the three PM-MRD sub-groups, since with an increasing PM-MRD, the median WBC-MRD will also increase (WBC-MRD being the product of PM-MRD and PM%). In our study, most WBC-MRD values were found to be low in the PM-MRD group (<2.34%), and most WBC-MRD values were high in the poor prognosis PM-MRD group (≥10%), while intermediate values are found in the intermediate PM-MRD group. However, as we outlined previously, the WBC-MRD levels for individual patients might be skewed by the PM%, especially at high and low PM% values. 

The total tumor load in solid tumors, both before and especially after therapy, has a direct relationship with patient prognosis [46]. For AML, primitive AML cells (progenitors and/or stem cells), when expressed as the total load (per WBC), have been shown to have prognostic impact [36,38,39,47,48]. To our knowledge, the present paper shows, for the first time, that, in AML, it is not the total tumor burden (WBC-MRD) that is most important for clinical outcome but the contribution of AML progenitors to the total progenitor compartment or, in other words, the balance between tumor and normal progenitor cells. It is therefore tempting to speculate that, at a certain threshold, the fast growth of acute leukemic progenitors [32,33,34], may overrule initial differences in the total tumor load (i.e., dividing plus non-dividing AML cells as percentages of WBC). 

Apart from this new insight into how to define residual disease, an advantage of the PM-MRD approach is that it circumvents most subjective elements of MRD identification, while less experience is required to define CD34+, CD117+, and CD133+ cells and aberrancies thereon than to define the extent to which normal bone marrow differentiation patterns have become aberrant in AML. A final advantage of the PM-MRD approach is that it circumvents the use of the total progenitor load (PM%, i.e., CD34 and/or CD117 and/or CD133 as a percentage of WBC) and therefore also the WBC load. The uncertainty of the WBC count, either resulting from the gating procedure of all viable WBC as such, or hemodilution, or as a result of selective degradation of WBC sub-populations (e.g., granulocytes) upon sample storage or transport [22,26] will affect correct WBC-MRD quantitation and thus make PM-MRD a potentially more reliable measure for residual disease quantitation. We have already shown this in a small study where the frequency of MRD in peripheral blood was much lower than in bone marrow, while the AML fraction of the primitive compartment was the same in peripheral blood and bone marrow [35]. It would be interesting to see whether this can be confirmed using several studies in which bone marrow MRD and peripheral blood MRD have already been compared [49,50,51,52].

As outlined in the Materials and Methods section, in most cases, CD133 may be replaced by CD117, thereby allowing us to monitor PM-MRD with only CD34 and CD117 as primitive markers, markers that are used in practice for MRD assessment in most institutes.

We have also shown that the identification of progenitors and aberrancies on it is possible with a different-from-normal approach, especially when no diagnostic material is available. In addition, the PM-MRD approach, in comparison with the diagnosis LAIP approach, allows the identification of new upcoming progenitor populations and therefore disappearing populations as well [29,30,31]. The additional advantages of PM-MRD discussed above may make its use important, especially in multicenter clinical studies aimed at the refinement of risk assessment, to guide follow-up therapy or for use as a short-term endpoint for survival. It therefore has to be figured out if more up-to-date aberrancy identification/quantification strategies are of additional advantage for PM-MRD [53].

Further studies need to be carried out in order to validate the prognostic value model of PM-MRD. As far as we know, no studies have reported on PM-MRD, but existing databases may contain the data needed to retrospectively extract PM-MRD, such as WBC-MRD% and PM% (CD34% or CD117%). If the PM% is not known, or if WBC-MRD contains more mature populations, a reanalysis of existing data is necessary to define PM-MRD. It was surprising to find that PM-MRD had prognostic impact in mature AML patients, since additional markers were thought to be necessary to define MRD (reviewed in [12]). However, more patients are needed to confirm this finding and to assess whether the three-group PM-MRD division is present in these cases too. Another recommendation for additional analysis concerns the PM-MRD region of 2.34–10%/MRD ≥ 0.1% (group V in Figure 4D). We provide evidence that this group of patients might represent a group with WBC-MRD false positivity due to its very high PM%. Again, this hypothesis needs to be confirmed in a larger patient group.

## 5. Conclusions

In conclusion, PM-MRD reveals the presence of three patient groups with different prognosis, but, in addition, also showed to represent the only prognostic part of WBC-MRD. As a first step, the approach to implement PM-MRD ≥ 10% as representative of the poor prognosis patient group, irrespective of MRD status (i.e., including both patients with WBC-MRD ≥ 0.1% and patients with WBC-MRD < 0.1%) would circumvent the occurrence of MRD false negatives in the present ELN-based MRD approach. Subsequently, the theory that a PM-MRD cut-off value can be used as a single parameter for residual disease prognostication, as presented in this paper, needs confirmation using retrospective studies of existing independent external databases and evaluation in parallel to MRD assessment in the upcoming clinical trials.

## Data Availability

The data presented in this study are available on request from the corresponding author. The data are not publicly available due to a pending HOVON data sharing policy.

## Supplementary Materials

The following are available online at https://www.mdpi.com/article/10.3390/cancers13112597/s1, Text S1: Rationale behind the equations, Text S2: Prognosis in PM-MRD subgroups sub-divided based on MRD, Figure S1: PM-MRD and WBC-MRD subdivided by cytogenetic risk group, Figure S2: PM-MRD in mature AML, Figure S3: PM% disturbs the relationship between MRD and EFS but not between PM-MRD and EFS, Figure S4: Relationship between WBC-MRD and PM-MRD in detail, Table S1: Primitive markers and LAIPs in the patient population, Table S2: Univariate testing of predictors for EFS, Table S3: Multivariate model of EFS in WBC-MRD and PM-MRD, Table S4: Cytogenetic and molecular characteristics of five WBC-MRD/PM-MRD combined subgroups, Table S5: Different-from-normal approach versus LAIP approach.
