# Supplementary material for: AML/Normal Progenitor Balance Instead of Total Tumor Load (MRD) Accounts for Prognostic Impact of Flowcytometric Residual Disease in AML
Source: Cancers (Basel). 2021 May 26;13(11):2597. doi: 10.3390/cancers13112597 (PMC8198261; doi:10.3390/cancers13112597)
Supplement: Supplementary file 1 [file cancers-13-02597-s001.zip › cancers-1232557-supplementary.pdf]

Supplementary Materials: AML/Normal Progenitor Balance Instead of Total Tumor Load (MRD) Accounts for Prognostic Impact of Flowcytometric Residual Disease in AML

Diana Hanekamp, Jesse M. Tettero, Gert J. Ossenkoppele, Angèle Kelder, Jacqueline Cloos and Gerrit Jan Schuurhuis

Text 1: Rationale behind the equations

In the present paper, the term MFC-MRD is used to define residual disease in the classical way (golden standard) of identifying and quantifying aberrant populations, referred to by us as LAIP+ populations, as percentages of all WBC. So, MFC-MRD = LAIP$^+$ cells/All WBC. These aberrant cells may be both progenitor cells (CD34+ or CD117+ or CD133+) and more mature populations. So,

MFC-MRD = (LAIP$^+$ progenitors + LAIP$^+$ non-progenitors)/WBC.

In our institute, under MRD conditions, we used LAIP+ progenitors as the basis for MRD, since we have found that when using progenitors, few MRD negative patients became MRD positive when more mature populations were incorporated. Our present approach is termed WBC-MRD and is defined as WBC-MRD = LAIP$^+$ progenitors/WBC.

As indicated in the main text (Materials and Methods), the prognostic impact of WBC-MRD was the same as that presented previously [1]; herein, in some cases, WBC-MRD was corrected for the partial coverage of blast cells with LAIP at diagnosis. This approach has revealed the best prognostic impact for WBC-MRD throughout the years [1–3] and was therefore compared with the newly defined PM-MRD.

When not corrected for coverage with LAIP at diagnosis, ("WBC-MRD$^{\text{not corrected}}$"), MRD levels were lower in some patients with a consequent decrease in the number of patients in group III of Figure 4D from 44 to 36 and a concomitant increase in group IV from 23 to 31 patients (see also legends of Figure 4D). Since this is just a shift between groups III and IV, which have similarly poor prognoses, this again emphasizes that the characteristic of PM-MRD ≥ 10% is enough to identify all patients with a poor prognosis independent of the MRD cut-off value. In parallel, the number of patients in group V decreased from 20 to 12.

As an example of calculations, the bone marrow aspirate of a particular patient may consist of a CD34+ population at diagnosis, which, is completely (100%) covered with LAIP. In the follow up of this patient, 30% (0.3 part) of the CD34+ population was found to be defined by the LAIP (so PM-MRD = 0.3). With a total (AML + normal) PM% of, e.g., 0.5 (% of WBC; PM%), WBC-MRD = 0.3 part of 0.5% = 0.15% (% of WBC). In cases in which LAIP coverage at diagnosis was only 60%, the WBC-MRD was corrected for this in the Zeijlemaker approach if there was good evidence that the non-LAIP+ rest of the blast population at diagnosis was also neoplastic. The WBC-MRD was then 100/60 × 0.15% = 0.25%. If, in the latter case, no correction was applied for this 60% coverage at diagnosis, when using the pure LAIP+ population, the WBC-MRD$^{\text{not corrected}}$ would be 0.3 part of 0.5% = 0.15%.

Note that it is only the definition of WBC-MRD (so, focused on progenitors) that allows us to quantitatively establish the contributions of both PM% and PM-MRD to the prognostic impact separately. Having non-progenitors in the MRD definition, as is the case for classical MFC-MRD, would not allow this approach.

Text 2: Prognosis in PM-MRD Subgroups sub-Divided based on MRD

WBC-MRD levels in the whole PM-MRD≥10% poor prognosis (III+IV) group show large heterogeneity with levels ranging from 0.007% to 9% (factor > 1000, best seen in Figure S4). This is due to the very large range of PM% values (factor >> 1300) in the PM-MRD area between 10% and 100% (Figure 4C). It can therefore be assumed that, apart from the WBC-MRD false negativity described in Figure 4D, some of the patients may be characterized by false positivity (e.g., if WBC-MRD > 5%, but the patient is still in remission). The latter would, however, not have an effect on present clinical decision making, since all patients here already have MRD values above the ELN cut-off (MRD ≥ 0.1%).

Because false negativity and false positivity can have clinical consequences, for the analysis presented below, we used the terms "artificially" low or "artificially high" WBC-MRD values, respectively, referring to the effects seen with extreme PM-percentages (low versus high, respectively).

Although the absence of a prognostic impact for the PM% (Figure 4A,B) already shows that its incorporation (together with PM-MRD) in the final definition of WBC-MRD has no advantage for defining prognosis compared with using PM-MRD alone, we chose to investigate this in a more direct way by assessing whether there are any effects of WBC-MRD on EFS in areas where EFS, as defined by PM-MRD alone, is relatively constant. In that way, we predicted that a possible additional effect of WBC-MRD (with its huge ranges of values) on EFS would become evident. Useful areas were PM-MRD 0–2.34%, 2.34–10%, and 10–70% (except for 10 patients with PM-MRD≥70%, where also most WBC-MRD values are >5%, where EFS was very poor: 0%). In each of these areas, there was a very high level of heterogeneity for WBC-MRD values (Figure S4A). In order to obtain relevant prognostic data, the WBC-MRD based patient sub-groups in each of these areas needed to comprise at least 30–40 patients.

In the group with PM-MRD 10–70%, two WBC-MRD-based equal patient groups were thus arbitrary defined: WBC-MRD 0–0.23% ($n = 28$) and WBC-MRD ≥ 0.23% ($n = 29$). Between these groups, there was no difference in EFS: 39% and 42%, respectively (Figure S4B).

There were very large ranges of WBC-MRD in both sub-groups (see Figure 4D and Figure S4A). The absence of EFS differences between the two groups strongly suggests that patients in the WBC-MRD < 0.23% group (thanks to the PM% term) are likely to have artificially low WBC-MRD values (this includes the MRD false negative group IV). Patients in the WBC-MRD ≥ 0.23% group (thanks to the PM% term) are likely to have artificially high WBC-MRD values (part of whom with WBC-MRD > 5%).

In the group with PM-MRD 2.34–10%, we defined three WBC-MRD based patient sub-groups (all with 41 patients): WBC-MRD < 0.0169%, WBC-MRD 0.0169%-0.039%, and WBC-MRD ≥ 0.039%). Again, there was no significant difference in EFS between the three groups: 42.9% versus 54.6% versus 50.5% (Log-Rank $p = 0.743$) (Details in Figure S4C).

The group with WBC-MRD < 0.0169 and that with WBC-MRD ≥ 0.039% showed large amounts of spreading, similar to the two WBC-MRD sub-groups defined above for the PM-MRD 10–70% region. This, again, suggests that these groups contain patients with artificially low and artificially high WBC-MRD values. In agreement with this, patients in sub-group V, who have high to very high WBC-MRD values (0.1–about 2%), do not seem to result in a poorer prognosis of the WBC-MRD ≥ 0.039% group in which they reside. Since group V represents patients with intermediate PM-MRD based prognoses but with relatively high WBC-MRD values resulting from high PM% values, we studied whether there were obvious differences between group V and the other groups, such as cytogenetics. No significant differences were found between V and all other sub-groups (Supplementary Table IV).

Similarly, in the group PM-MRD 0–2.34%, division into two patient groups (both 55 patients) resulted in a group with WBC-MRD < 0.0074% with an EFS of 65% and a group with WBC-MRD ≥ 0.0074% with an EFS of 63% (Log-Rank $p = 0.876$) (Figure S4D). It has to be mentioned that, at these relatively low PM-MRD and WBC-MRD values, the values

are not always accurate, especially when PM-MRD values approach normal bone marrow background values.

In conclusion, these analyses show that over the large PM-MRD range of 0–70%, the widely different WBC-MRD values suggest that there are no additional effects of WBC-MRD on the prognostic value delivered by PM-MRD alone. Moreover, they cause artificially high and artificially low WBC-MRD values in some patients, potentially leading to the use of MDR false-positivity and MRD false negativity MRD cut-off values in the clinic.

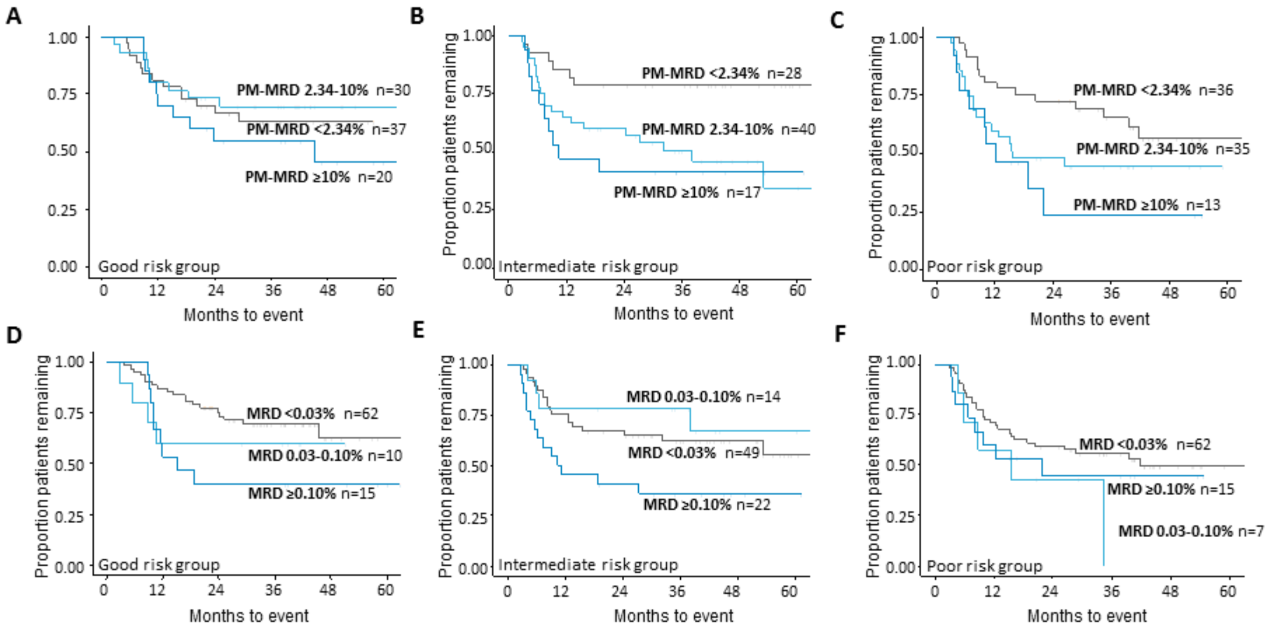

Figure S1: PM-MRD and WBC-MRD subdivided by cytogenetic risk group. (**A**) PM-MRD Good risk group (n = 87), (**B**) PM-MRD Intermediate risk group (n = 85) and (**C**) PM-MRD Poor risk group (n = 84). (**D**) WBC-MRD Good risk group (n = 87), (**E**) WBC-MRD Intermediate risk group (n = 85) and (**F**) WBC-MRD Poor risk group (n = 84).

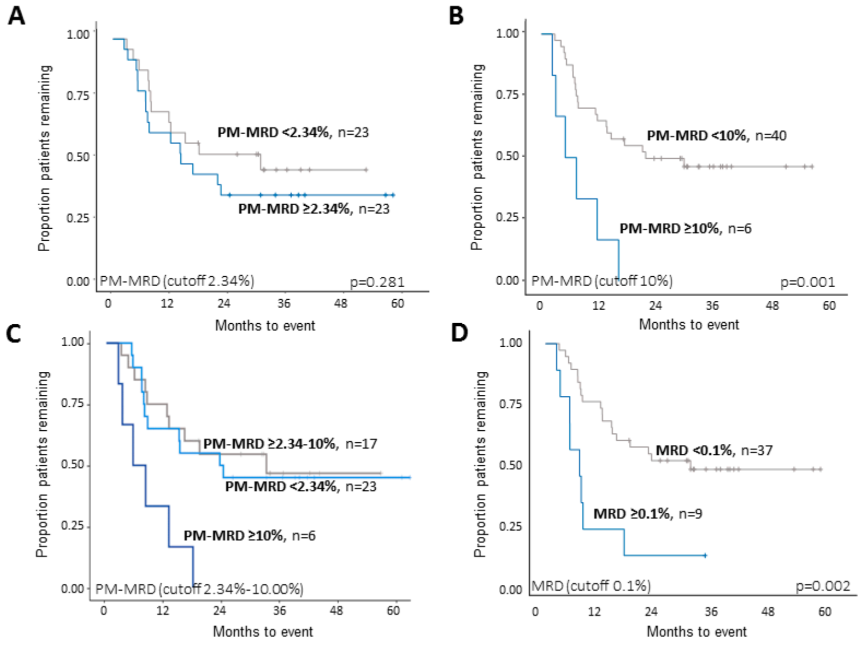

Figure S2. PM-MRD and WBC-MRD in mature AML. In our cohort, 46 patients (15.3% of the 300 patients) had more mature AML, here classified as French–American–British (FAB) classifications M5 (n = 38), M6 (n = 6) or M7 (n = 2). (**A**)–(**C**): PM-MRD cut-offs, similar to the whole patient group (Figure 2) may define patients with different prognosis. (**D**) For reference, MRD with a cut-off of 0.1% (19.5 % of total number) is shown in (**D**).

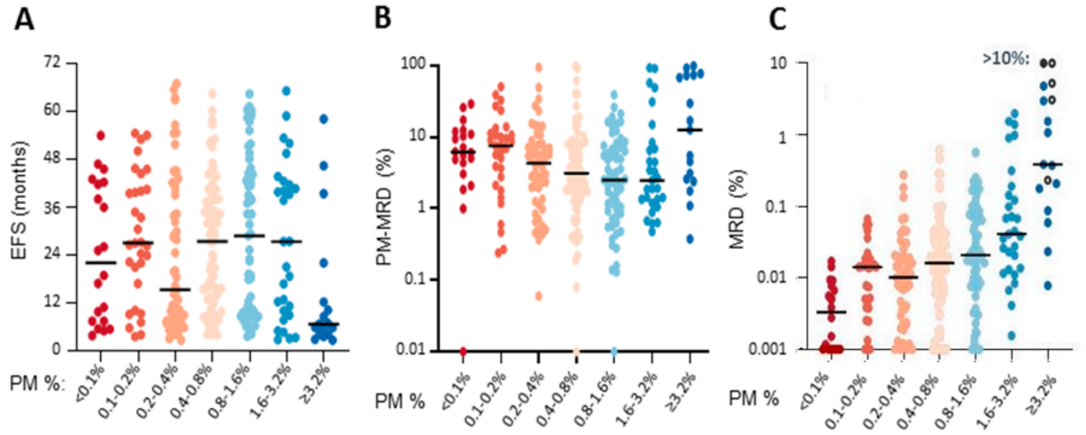

Figure S3. PM% disturbs the relationship between WBC-MRD and EFS but not between PM-MRD and EFS. The PM% was subdivided in the following groups, <0.1% (*n* = 20), 0.1–0.2% (*n* = 31), 0.2–0.4% (*n* = 55), 0.4%-0.8% (*n* = 87), 0.8–1.6% (*n* = 61), 1.6%-3.2% (*n* = 29) and ≥3.2% (*n* = 17). (**A**) Although Figure 4A,B is best used for comparison between PM% and EFS (censoring was namely applied for those figures), we used this Figure S3A because it allows a direct comparison with the B and C panels, described below. In (A), the actual EFS value for each individual patient is shown in the different PM% sub-groups. Note the large spreading in each sub-group, and compatible with the Figures 4A,B, the minor differences in median EFS in all sub-groups, except the PM ≥ 3.2% sub-group. In the 0.2–0.4% group there was an unexplained lower EFS compared to the other groups. (**B**) PM-MRD for each individual patient in the different PM% sub-groups. Overall PM-MRD was the same in all sub-groups except the PM% ≥ 3.2%. PM-MRD thus correlated with EFS: compare (**B**) with (A). (**C**) WBC-MRD for each individual patient in the different PM% sub-groups. Note the large spreading in each sub-group. This kind of analysis, however, also revealed that, with the increasing PM percentages, an increase in of median WBC-MRD (at least a factor 10 from PM 0%–PM 3.2% (Pearson corr. coefficient 0.367, $p < 0.0001$; Spearman test corr. coefficient 0.388, $p < 0.0001$) is not paralleled by a decrease of EFS: compare (**C**) with (**A**). WBC-MRD thus not always correlates with EFS.

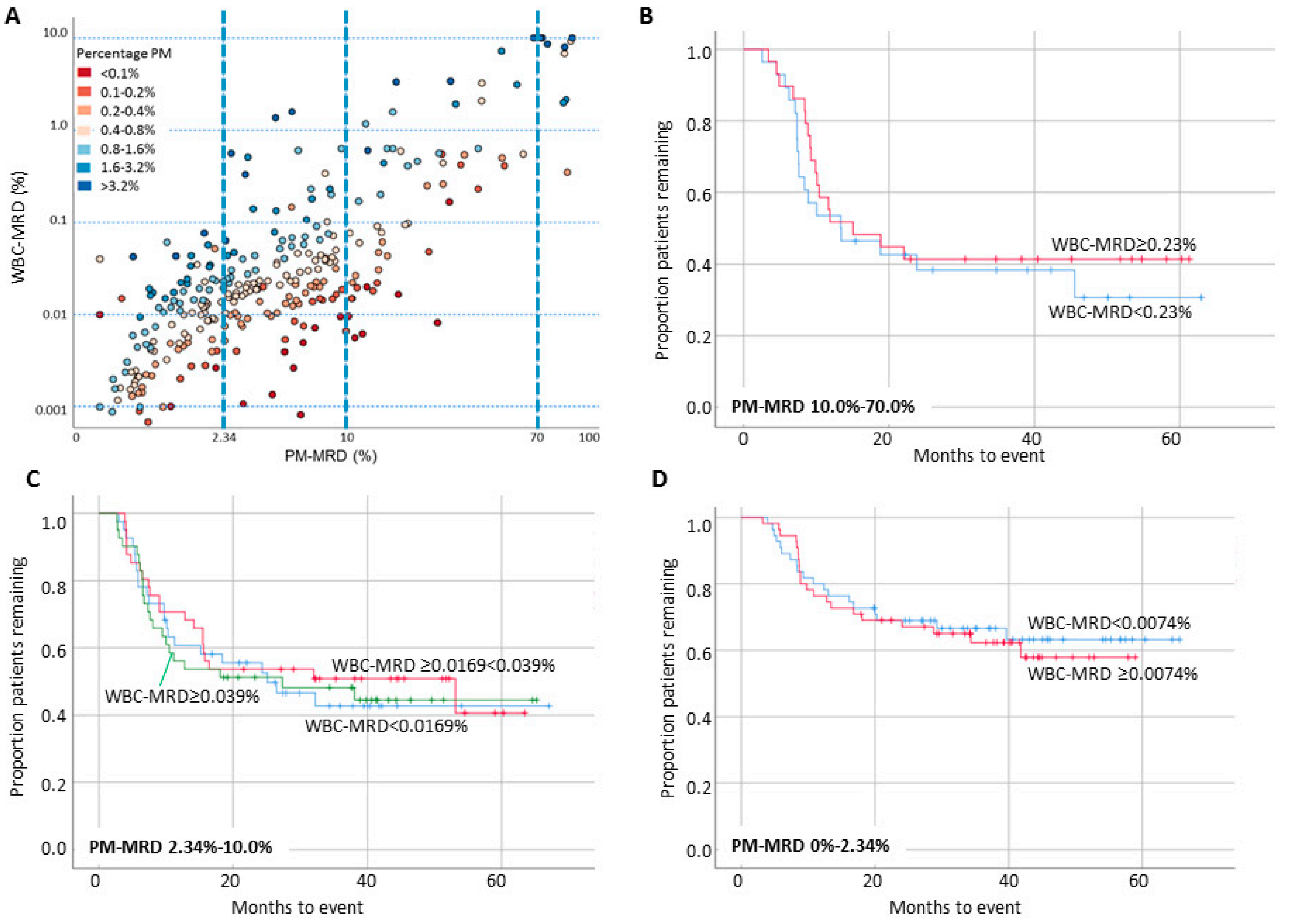

Figure S4: Relationship between WBC-MRD and PM-MRD for individual patients and consequences for EFS in sub-groups. (**A**) The figure is a blow up of Figure 3D with both axes represented logarithmically. It showed in detail how the product of PM-MRD and PM% resulted in large heterogeneity of WBC-MRD in all PM-MRD regions. The figure illustrates that over the whole range of PM-MRD values, there was a huge heterogeneity of corresponding WBC-MRD values, often exceeding factor 1000 in the three different PM-MRD regions that were defined before in Figure 4D (here defined by three thicker dotted lines). As outlined in Supplementary Text 2, dividing each of the three PM-MRD regions (defined in Figure 4D) in two or three equal sub-regions based on WBC-MRD, did not result additional prognostic impact of WBC-MRD: (**B**) for PM-MRD 10–70% (PM-MRD ≥ 70% (n = 10) was excluded here because EFS was 0%); (**C**) for PM-MRD 2.34–10%; (**D**) for PM-MRD 0–2.34%.

Table S1. Primitive markers and LAIPs in the patient population

PM	LAIP	MRD in Patients Median (Range)	PM-MRD in Patients Median (Range)	Cases (Number)
**CD34**	CD7+	0.03 (0.00–0.99) 0.03 (0.00–	7.14 (0.60–91.71) 4.50	47
	CD56+	23.0)	(0.16–77.6)	30
	CD33−C13+	0.01 (0.00–0.20)	2.60 (0.37–32.75)	26
	CD13−CD33+	0.02 (0.01–0.02)	2.53 (0.93–10.02)	5
	CD13−CD117+	0.19 (0.14–0.24)	17.03 (6.91–27.14)	2
	CD19+	1.00 (0.01–1.98)	50.12 (9.45–90.79)	2
	CD15+	0.09 (0.00–0.28)	15.49 (0.22–61.05)	14
	CD22+	0.01 (0.00–0.92)	10.42 (1.06–31.10)	8
	CD14+	0.02 (0.02–0.02)	3.67 (3.67–3.67)	1
	CD11b+	0.02 (0.00–1.54)	2.96 (0.51–94.42)	25
	CD2+	0.03 (0.01–0.04)	5.36 (0.00–10.71)	2
	HLADR−	0.01 (0.00–0.09)	1.23 (0.14–13.94)	14
**CD117**	CD7+	0.03 (0.00–5.28)	4.49 (1.02–97.62)	33
	CD56+	0.03 (0.01–1.56)	5.49 (0.38–26.29)	12
	CD33−CD13+	0.01 (0.01–0.01)	0.78 (0.78–0.78)	1
	CD13−CD33+	0.01 (0.00–0.04)	1.80 (0.31–8.62)	6
	CD19+			0
	CD22+	0.02 (0.02–0.02)	5.33 (2.77–7.89)	2
	CD14+			0
	CD11b+	0.01 (0.00–0.12)	2.70 (0.70–6.84)	9
	CD2+	0.00 (0.00–0.00)	0.85 (0.85–0.85)	1
	CD15-HLADR−	0.01 (0.00–27.9)	1.91 (0.28–99.06)	31
**CD133**	CD34−	0.01 (0.00–0.24)	1.93 (0.00–39.02)	28

Table S2. Univariate testing of predictors for EFS.

Variables		Coefficient	SE	95% CI	*p* Value
Sex	Female	0.726	0.165	0.526–1.003	0.052
Age	Per year	1.014	0.007	0.999–1.029	0.062
WBC-count × 10^9 L	20				
	20-100	1.849	0.189	1.278–2.675	0.001
	100	2.180	0.265	1.298–3.661	0.003
HOVON risk group	Good				
	Intermediate	1.329	0.242	0.828–2.134	0.239
	Poor	1.580	0.235	0.998–2.503	0.051
	Very poor	4.275	0.243	2.654–6.886	0.000
Cycles before CR	2 Cycles vs 1 cycle	2.473	0.201	1.668–3.667	0.000
Flow MRD	MRD 0.03	1.679	0.164	1.217–2.317	0.002
	MRD 0.1	1.753	0.183	1.226–2.507	0.002
	PM-MRD 2.34	1.965	0.185	1.367–2.825	0.000
	PM-MRD 10.0	1.763	0.180	1.239–2.510	0.002
CD34status DX	CD34pos				
	CD34pos, no LSC	0.939	0.390	0.448–0.876	0.872
	CD34neg	0.792	0.244	0.380–1.777	0.339
Arm H102	Standard				
	Clofarabine 10mg	0.627	0.171	0.448–0.876	0.006
	Clofarabine 15mg	0.821	0.394	0.380–1.777	0.617
HSCT type	Allogeneic				
	Autologous	1.137	0.241	0.709–1.822	0.595
PM percentage	0.1				
	0.1–0.2	0.599	0.418	0.264–1.359	0.220
	0.2–0.4	1.050	0.353	0.526–2.096	0.890
	0.4–0.8	0.798	0.338	0.411–1.547	0.504
	0.8–1.6	0.735	0.358	0.364–1.482	0.389
	1.6–3.2	0.710	0.417	0.313–1.608	0.411

	3.2	2.588	0.404	1.173–5.709	0.018

Table S3 (**A**). Multivariate model EFS / PM-MRD 2.34% cut-off. (**B**). Multivariate model EFS / PM-MRD 10.0% cut-off. (**C**). Multivariate model EFS / WBC-MRD 0.03% cut-off. (**D**). Multivariate model EFS / WBC-MRD 0.1% cut-off.

A

Variables		Coefficient	SE	95% CI	*p* Value
PM-MRD 2.34		1.828	0.191	1.258–2.655	0.002
WBC-count × 10^9 L	20				
	20-100	2.087	0.197	1.418–3.072	0.000
	100	2.781	0.278	1.614–4.793	0.000
HOVON risk group	Good				
	Intermediate	1.395	0.247	0.860–2.262	0.177
	Poor	1.472	0.259	0.886–2.445	0.135
	Very poor	3.953	0.257	2.388–6.545	0.000
Cycles before CR	2 Cycles vs 1 cycle	2.065	0.233	1.307–3.263	0.002
Arm H102	Standard				
	Clofarabine 10mg	0.643	0.174	0.457–0.904	0.011
	Clofarabine 15mg	1.018	0.398	0.467–2.219	0.964

B

Variables		Coefficient	SE	95% CI	*p* Value
PM-MRD 10.0		1.582	0.188	1.058–2.208	0.024
WBC-count x 10^9 L	20				
	20–100	2.036	0.197	1.383–2.996	0.000
	100	2.682	0.282	1.542–4.664	0.000
HOVON risk group	Good				
	Intermediate	1.489	0.247	0.917–2.419	0.107
	Poor	1.436	0.260	0.862–2.391	0.164
	Very poor	4.060	0.256	2.456–6.710	0.000
Cycles before CR	2 Cycles vs 1 cycle	2.119	0.232	1.345–3.338	0.001
Arm H102	Standard				
	Clofarabine 10mg	0.638	0.175	0.453–0.898	0.010
	Clofarabine 15mg	1.033	0.399	0.473–2.256	0.935

C

Variables		Coefficient	SE	95% CI	*p* Value
WBC-MRD 0.03		1.384	0.172	0.967–1.897	0.078
WBC-count x 10^9 L	20				
	20–100	2.092	0.197	1.421–3.080	0.000
	100	2.494	0.277	1.448–4.294	0.001
HOVON risk group	Good				
	Intermediate	1.341	0.248	0.825–2.179	0.237
	Poor	1.394	0.257	0.842–2.308	0.196
	Very poor	3.835	0.262	2.294–6.412	0.000
Cycles before CR	2 Cycles vs 1 cycle	2.129	0.232	1.351–3.355	0.001
Arm H102	Standard				
	Clofarabine 10mg	0.667	0.175	0.473–0.940	0.021
	Clofarabine 15mg	1.047	0.399	0.479–2.287	0.908

D					
**Variables**		**Coefficient**	**SE**	**95% CI**	***p* Value**
WBC-MRD 0.1		1.518	0.188	1.050–2.193	0.026
WBC-count x 10^9 L	20				
	20-100	2.122	0.197	1.442–3.122	0.000
	100	2.651	0.281	1.529–4.598	0.001
HOVON risk group	Good				
	Intermediate	1.402	0.246	0.865–2.272	0.170
	Poor	1.378	0.259	0.830–2.288	0.215
	Very poor	3.994	0.257	2.415–6.605	0.000
Cycles before CR	2 Cycles vs 1 cycle	1.518	0.188	1.050–2.193	0.026
Arm H102	Standard				
	Clofarabine 10mg	2.122	0.197	1.442–3.122	0.000
	Clofarabine 15mg	2.651	0.281	1.529–4.598	0.001

Table S4. Cytogenetic and molecular characteristics of five WBC-MRD/PM-MRD combined subgroups. In order to investigate differences between group V and other groups, Chi-square test (or Fischer exact test if a cell contained less than five patients) were conducted to compare both groups. No significant differences between group V and other groups were found (all $p > 0.05$).

Variables		I		II		III		IV		V		Total	
		Count	Column *n* %	Count	Column *n* %	Count	Column *n* %	Count	Column *n* %	Count	Column *n* %	Count	Column *n* %
Sex	Male	46	42,2%	55	52,9%	19	43,2%	14	60,9%	13	65,0%	147	49,0%
	Female	63	57,8%	49	47,1%	25	56,8%	9	39,1%	7	35,0%	153	51,0%
WBC-count × 10^9 L	20	76	69,7%	75	72,1%	27	61,4%	15	65,2%	15	75,0%	208	69,3%
	20–100	21	19,3%	22	21,2%	15	34,1%	7	30,4%	3	15,0%	68	22,7%
	100	12	11,0%	7	6,7%	2	4,5%	1	4,3%	2	10,0%	24	8,0%
HOVON risk group	Good	37	33,9%	27	26,0%	12	27,3%	8	34,8%	3	15,0%	87	29,0%
	Intermediate	28	25,7%	31	29,8%	13	29,5%	4	17,4%	9	45,0%	85	28,3%
	Poor	36	33,0%	30	28,8%	10	22,7%	3	13,0%	5	25,0%	84	28,0%
	Very Poor	8	7,3%	16	15,4%	9	20,5%	8	34,8%	3	15,0%	44	14,7%
CD34 status at diagnosis	CD34 pos	72	75,8%	70	76,1%	28	82,4%	20	90,9%	16	88,9%	206	78,9%
	CD34 pos, no LSC	3	3,2%	6	6,5%	3	8,8%	0	0,0%	1	5,6%	13	5,0%
	CD34 neg	20	21,1%	16	17,4%	3	8,8%	2	9,1%	1	5,6%	42	16,1%
t(8;21)	neg	86	84,3%	88	91,7%	35	81,4%	21	100,0%	18	94,7%	248	88,3%
	pos	13	12,7%	6	6,3%	5	11,6%	0	0,0%	1	5,3%	25	8,9%
	ND	3	2,9%	2	2,1%	3	7,0%	0	0,0%	0	0,0%	8	2,8%
inv(16)	neg	95	93,1%	90	93,8%	36	83,7%	20	95,2%	18	94,7%	259	92,2%
	pos	4	3,9%	3	3,1%	5	11,6%	1	4,8%	1	5,3%	14	5,0%
	ND	3	2,9%	3	3,1%	2	4,7%	0	0,0%	0	0,0%	8	2,8%
FLT3-ITD	neg	72	72,0%	68	73,1%	28	71,8%	19	90,5%	12	63,2%	199	73,2%
	pos	28	28,0%	25	26,9%	11	28,2%	2	9,5%	7	36,8%	73	26,8%
NPM1	neg	67	62,6%	68	66,0%	31	73,8%	18	78,3%	13	65,0%	197	66,8%
	pos	40	37,4%	35	34,0%	11	26,2%	5	21,7%	7	35,0%	98	33,2%
FLT3-ITD x NPM1	FLT3ITD neg NPM1 neg	50	50,0%	54	58,1%	24	63,2%	14	66,7%	11	57,9%	153	56,5%

	FLT3ITD pos NPM1 neg	15	15,0%	9	9,7%	4	10,5%	2	9,5%	2	10,5%	32	11,8%
	FLT3ITD neg NPM1 pos	22	22,0%	14	15,1%	3	7,9%	5	23,8%	1	5,3%	45	16,6%
	FLT3ITD pos - NPM1 pos	13	13,0%	16	17,2%	7	18,4%	0	0,0%	5	26,3%	41	15,1%
CEBPA mutation (double)	neg	85	97,7%	69	94,5%	30	93,8%	16	94,1%	18	100,0%	218	96,0%
	pos	2	2,3%	4	5,5%	2	6,3%	1	5,9%	0	0,0%	9	4,0%
EVI1 over-expression	neg	84	95,5%	73	90,1%	30	83,3%	16	84,2%	17	94,4%	220	90,9%
	pos	4	4,5%	8	9,9%	6	16,7%	3	15,8%	1	5,6%	22	9,1%

Table S5 (**A**). Different-from-normal versus LAIP at diagnosis approaches. WBC-MRD and PM-MRD. (**B**). Different from normal approach for progenitors identifying MRD and PM-MRD in the absence of diagnosis LAIPs.

A

**Patient**	**Diagnosis LAIP (all LAIPs CD34+)**	**WBC-MRD based on Diagnosis LAIPs**	**PM-MRD based on Diagnosis LAIP**	**FU Blindly Analyzed for LAIPs**	**WBC-MRD based on FU LAIP**	**PM-MRD based on FU LAIP**
A	No LAIP			No LAIP		
B	CD15	0.12%	8.3%	CD15<br>CD22	0.1%<br>0.09%	6.9%<br>5.8%
C	No LAIP			No LAIP		
D	CD7	0.13%	7.4%	CD7<br>CD2	0.23%<br>0.08%	14.4%<br>4.6%
E	No LAIP			No LAIP		
F	No LAIP			No LAIP		
G	No LAIP			No LAIP		
H	No LAIP			Relapse		
I	CD7	0.41%	8.1%	CD7<br>CD2	0.46%<br>0.18%	10.4%<br>4.4%
J	No LAIP			CD11b<br>CD56<br>CD22<br>CD33++<br>HLA-DR++	0.14%<br>0.08%<br>0.05%<br>0.14%<br>0.49%	23.2%<br>13.7%<br>8.9%<br>22.6%<br>82.1%
K	No LAIP			No LAIP		
L	No LAIP			No LAIP		
M	No LAIP			No LAIP		
N	CD7<br>CD19	0.02%<br>0%	3.8%<br>0.74%	CD56<br>CD15	0.13%<br>0.11%	26.5%<br>23.4%
O	CD7<br>CD2<br>CD56<br>CD11b	1.43%<br>5.69%<br>3.38%<br>0.25%	15.7%<br>62.7%<br>37.0%<br>2.8%	CD7<br>CD2<br>CD56<br>CD11b<br>HLA-DR-	2.35%<br>5.43%<br>5.54%<br>1.01%<br>3.59%	21.6%<br>51.4%<br>51.1%<br>9.6%<br>32.7%
p	No LAIP			No LAIP		

B

A	FU3	CD22<br>HLA-DR-	2.77%<br>0.76%	12.3%<br>3.36%

B	FU1	CD7	0.1%	6.9%
		CD15	0.09%	5.8%
	FU2	CD7	2.25%	7.8%
		CD15	1.52%	5.3%
	FU3	CD7	0.57%	21.5%
	FU4	CD7	0.06%	7.0%
	FU5	CD7	0.11%	11.3%
		CD15	0.03%	3.6%
C	FU1	CD56	0.99%	31.0%
	FU2	CD56	0.04%	4.4%
	FU3	CD56	0.01%	1.5%
	FU4	CD56	0.14%	1.5%
	FU5	CD56	0.18%	0.7%
D	FU2	CD56	0.58%	19.1%

In order to prevent bias, the cases shown are from a short time period of MRD assessments in the HOVON102 study, without exclusion of samples. All samples were from bone marrow, with the exception of patient 2 which was taken from leukaferesis material. Columns 3 and 4 show WBC-MRD and PM-MRD based on the Diagnosis LAIPs as assessed in column 2. Columns 5, 6 and 7 revealed both LAIPs, WBC-MRD and PM-MRD, respectively, assessed without knowledge of Diagnosis LAIPs. FU: at a clinical follow up time point (usually after the second induction course). Note that in those cases with diagnosis LAIPs present, the blind FU investigation identified the same follow up LAIPs as in the direct diagnosis LAIP approach (which were shown in columns 2–4), except for patient N, in which, however, new LAIPs were identified at FU. Note also that in FU additional LAIPs were identified using this different-from-normal progenitor approach (patient B, D, I, O, with patient even showing five LAIPs in the absence of diagnosis LAIPs). All flow cytometry tubes were used for each follow up (FU) sample. Data are from a parallel clinical study on elderly patients (HOVON103). From the four patients shown, two had more than one follow up time point.

References

1. Zeijlemaker, W.; Kelder, A.; Cloos, J.; Schuurhuis, G.J. Immunophenotypic Detection of Measurable Residual (Stem Cell) Disease Using LAIP Approach in Acute Myeloid Leukemia. *Curr Protoc Cytom* **2019**, *91*, e66, doi:10.1002/cpcy.66.
2. Feller, N.; van der Pol, M.A.; van Stijn, A.; Weijers, G.W.D.; Westra, A.H.; Evertse, B.W.; Ossenkoppele, G.J.; Schuurhuis, G.J. MRD parameters using immunophenotypic detection methods are highly reliable in predicting survival in acute myeloid leukaemia. *Leukemia* **2004**, *18*, 1380-–1390, doi:10.1038/sj.leu.2403405.
3. Terwijn, M.; van Putten, W.L.; Kelder, A.; van der Velden, V.H.; Brooimans, R.A.; Pabst, T.; Maertens, J.; Boeckx, N.; de Greef, G.E.; Valk, P.J.; et al. High prognostic impact of flow cytometric minimal residual disease detection in acute myeloid leukemia: data from the HOVON/SAKK AML 42A study. *J Clin Oncol* **2013**, *31*, 3889–3897, doi:10.1200/jco.2012.45.9628.
